# Protective Effects of *Taraxacum officinale* L. (Dandelion) Root Extract in Experimental Acute on Chronic Liver Failure

**DOI:** 10.3390/antiox10040504

**Published:** 2021-03-24

**Authors:** Iulia Olimpia Pfingstgraf, Marian Taulescu, Raluca Maria Pop, Remus Orăsan, Laurian Vlase, Ana Uifalean, Doina Todea, Teodora Alexescu, Corina Toma, Alina Elena Pârvu

**Affiliations:** 1 Department of Pathophysiology, Faculty of Medicine, Iuliu Haţieganu University of Medicine and Pharmacy Cluj-Napoca, 400012 Cluj-Napoca, Romania; cheta.iulia@umfcluj.ro (I.O.P.); uifaleanana@gmail.com (A.U.); parvualinaelena@umfcluj.ro (A.E.P.); 2 Department of Pathology, Faculty of Veterinary Medicine, University of Agricultural Sciences and Veterinary Medicine Cluj-Napoca, 400372 Cluj-Napoca, Romania; corina.toma@usamvcluj.ro; 3 Synevovet Laboratory, 021408 Bucharest, Romania; 4 Department of Pharmacology, Toxicology and Clinical Pharmacology, Iuliu Haţieganu University of Medicine and Pharmacy Cluj-Napoca, 400012 Cluj-Napoca, Romania; 5 Department of Physiology, Faculty of Medicine, Iuliu Haţieganu University of Medicine and Pharmacy Cluj-Napoca, 400012 Cluj-Napoca, Romania; remus.orasan@umfcluj.ro; 6 Department of Pharmaceutical Technology and Biopharmaceutics, Faculty of Pharmacy, Iuliu Haţieganu University of Medicine and Pharmacy Cluj-Napoca, 400012 Cluj-Napoca, Romania; laurian.vlase@umfcluj.ro; 7 Department of Pneumology, Faculty of Medicine, Iuliu Hatieganu University of Medicine and Pharmacy Cluj-Napoca, 400012 Cluj-Napoca, Romania; d.todea@umfcluj.ro; 8 4th Medical Clinic, Iuliu Hatieganu University of Medicine and Pharmacy Cluj-Napoca, 400012 Cluj-Napoca, Romania; teodora.alexescu@umfcluj.ro

**Keywords:** acute on chronic liver failure, hepatoprotective, oxidative stress, *Taraxacum officinale*, 3-nitrotyrosine

## Abstract

Background: *Taraxacum officinale* (TO) or dandelion has been frequently used to prevent or treat different liver diseases because of its rich composition in phytochemicals with demonstrated effect against hepatic injuries. This study aimed to investigate the possible preventing effect of ethanolic TO root extract (TOERE) on a rat experimental acute on chronic liver failure (ACLF) model. Methods: Chronic liver failure (CLF) was induced by human serum albumin, and ACLF was induced in CLF by D-galactosamine and lipopolysaccharide (D-Gal-LPS). Five groups (*n* = 5) of male Wistar rats (200–250 g) were used: ACLF, ACLF-silymarin (200 mg/kg b.w./day), three ACLF-TO administered in three doses (200 mg, 100 mg, 50 mg/kg b.w./day). Results: The in vivo results showed that treatment with TOERE administered in three chosen doses before ACLF induction reduced serum liver injury markers (AST, ALT, ALP, GGT, total bilirubin), renal tests (creatinine, urea), and oxidative stress tests (TOS, OSI, MDA, NO, 3NT). Histopathologically, TOERE diminished the level of liver tissue injury and 3NT immunoexpression. Conclusions: This paper indicated oxidative stress reduction as possible mechanisms for the hepatoprotective effect of TOERE in ACLF and provided evidence for the preventive treatment.

## 1. Introduction

Liver diseases are one of the major health problems in the world and became a general health care problem due to the high morbidity rate. They are associated with several risk factors such as inadequate nutrition, metabolic diseases, viral infection, ethanol, and drug use. Liver injury may trigger the onset of liver failure, a common medical condition with very high mortality [1]. Liver failure can progress as acute liver failure (ALF), as acute on chronic liver failure (ACLF), or as acute decompensation of end-stage liver disease [2]. ALF is defined as a severe liver injury in the absence of pre-existing liver disease. According to the World Gastroenterology Organization ACLF is defined as “a syndrome in patients with chronic liver disease with or without previously diagnosed cirrhosis, characterized by acute hepatic decompensation resulting in liver failure (jaundice and prolongation of the international normalized ratio) and one or more extrahepatic organ failures, associated with increased mortality up to three months” [2,3]. The prevalence of ACLF ranges from 24% to 40%, and it usually occurs in young or middle-aged patients [4] and it is potentially reversible [2]. 

The exact mechanism of ACLF is not fully elucidated but based on what was found the pathophysiology was described using a four-stage model: precipitating event, hepatic injury due to precipitating event, response to injury, and failure of other organs [4,5]. The precipitating event can be triggered by one or more factors, identified or unidentified, like infections, alcohol, gastrointestinal bleeding, reactivation of viral hepatitis B (HBV), superinfection with hepatitis A or E virus, acute episodes of autoimmune hepatitis, Wilson’s disease, or vascular liver disease [2,6,7]. During the propagation phase, the number of proinflammatory mediators increases, a systemic inflammatory response syndrome and a vascular endothelial dysfunction will be activated, with progression to organs failure. At the same time, liver macrophages release anti-inflammatory cytokines that will initiate a compensatory anti-inflammatory response syndrome, leading to an acquired immunodeficiency, a “paralysis of the immune response” [4,8]. Measurement of oxidative stress, inflammation, necrosis, and apoptosis biomarkers can define the risk profile of ACLF [4,8]. The presence of multiple organ failure is a requirement for the diagnosis of ACLF, and the number of affected systems has a prognostic value. The kidneys are the most commonly affected organs [2,3].

Thus, the therapy for chronic hepatic diseases needs to develop new prophylactic agents to prevent ACLF. With the extended studies upon the use of medicinal plants, phytotherapy became an important support for the treatment of many diseases. The use of medicinal plants in the treatment of liver diseases has a long history worldwide because many phytochemicals have hepatoprotective activity [1,9,10]. Only a few of the ethnomedicinal effects have been scientifically validated [11]. Considering that in ACLF inflammation and oxidative stress are important pathogenetic mechanisms, the herbal medicines that have anti-inflammatory and antioxidant effects could be a promising source of bioactive compounds [12].

The *Taraxacum officinale* F. H. Wigg. (TO) (dandelion) species belong to the Asteraceae family, includes 30–57 varieties, and are widely distributed in the warm-temperate zones of the Northern Hemisphere [13,14,15]. It is a plant used in folk medicine from ancient times as anti-inflammatory, antioxidant, diuretic, choleretic, laxative, and hepatoprotective. Because the phytochemical components may define the medicinal value of a plant, their identification and effects mechanism in disease prevention and treatment is a necessity [11]. Furthermore, it has to be considered that the chemical composition of the TO extracts depends on both the extraction protocol and the solvents used (ethanol, acetone, water, or methanol) [16], but also on which part of the plant has been used (whole plant, roots, stem, leaves, flowers). 

TO is also frequently used in different food products, and dietary supplements [17]. These plants were found to be rich in polyphenolic compounds, vitamins, inositol, lecithin, and minerals, and to exhibit antioxidant, anti-inflammatory, antiallergic, anti-hyperglycemic, hypolipidemic, and anticoagulant activities [9,18,19], to protect against hepatic injuries, but the mechanisms of action are still incompletely investigated [20]. 

It was demonstrated that TO root extract may protect against some toxic hepatic injury [11,13,21,22], but there are no studies on the potential hepatoprotective effect of this extract in ACLF. Therefore, our study aimed to extend the characterization of the ethanolic TO root extract (TOERE) and evaluate the potential use as a preventive hepatoprotective agent in a rat d-galactosamine and lipopolysaccharide (D-Gal-LPS)-induced rat ACLF model.

## 2. Materials and Methods

### 2.1. Chemicals

Phenolic compounds Sigma (St. Louis, MO, USA), Roth (Karlsruhe, Germany), Dalton (Toronto, ON, Canada); phytosterols Sigma (St. Louis, MO, USA); Folin–Ciocâlteu reagent, sulfanylamide (SULF), N- (1-Naphthyl) ethylenediamine dihydrochloric acid (NEDD), vanadium chloride (III) (VCl3), methanol, diethyl ether, xylenol orange [o-cresosulfonphthalein-3,3-bis (sodium methyliminodiacetate)], orthodianisidinedihydrochloric acid (3-3′-dimethoxybenzidine), ferrous ammonium sulfate, hydrogen peroxide (H2O2), sulfuric acid, hydrochloric acid, glycerol, trichloroacetic acid (TCA), ethylenediaminetetra-acetic acid, sodium dodecal, sulfate butylated hydroxytoluene, thiobarbituric acid, 1,1,3,3-tetraethoxypropane, 2,4-dinitrophenylhydrazine (DNPH), 5,5’-dithionitrobis 2-nitrobenzoic acid (DTNB), 1,1-diphenyl-2-picrilhydrazyl (DPPH), o-phthalaldehyde Merck (Darmstadt, Germany); Trolox (6-hydroxy-2,5,7,8-tetramethylchroman-2-carboxylic acid) Alfa-Aesar (Karlsruhe, Germany); Freund’s adjuvant, d-galactosamine (D-Gal) and lipopolysaccharide (LPS) from Merck and Sigma-Aldrich (Taufkirchen, Germany); Human serum albumin (HSA) (Octapharma GmbH, Austria). All chemicals were of analytical grade. Aspartate aminotransferase, alanine aminotransferase, total bilirubin, alkaline phosphatase, gamma glutamate transferase, creatinine, and urea kits were purchased from Spinreact (Sant Esteve de Bas, Spain). ELISA kit for 3-nitrotyrosine (KA0445-ABNOVA EMBLEM, Heidelberg, Germany) and primary antibody to 3-Nitrotyrosine for immunohistochemistry (Code ALX-804-505-C050, Enzo Life Sciences) were also used.

### 2.2. Plant Material

Fresh *T. officinale* F.H. Wigg. roots from the Alexandru Borza Botanical Garden “Babes-Bolyai” University of Cluj-Napoca, Romania, were purchased in June 2020, deposited in “Alexandru Borza” Botanical Garden Herbarium (Voucher CL:669002), and plant extract was prepared as previously described [23]. The roots were dried in a shaded place, grounded in a coffee grinder (Argis, RC-21, Electroarges SA, Curtea de Arges, Romania) for 5 min, and then the powder was screened through a 200 μm Retsch sieve [24]. Fifty grams were weighed and extracted with 70% ethanol, twice for 30 min using the UltraTurrax extraction apparatus (T 18; IKA Labortechnik, Staufen, Germany) at room temperature. The samples were then centrifuged at 4000 rpm for 30 min, and the supernatant was recovered, and filtered through a 0.45 μm micropore membrane (PTFE, Waters, Milford, MA, USA). The solvent was evaporated at 40 °C using a rotary evaporator (Hei-VAP, Heidolph Instruments GmbH & Co., Schwabach, Germany). Further, the obtained extracts were lyophilized (Advantage 2.0, SP Scientific, Warminster, PA, USA) [24]. The extract powder was stored at room temperature in airtight bottles. The extraction yield was 15.2% (*w/w*).

### 2.3. Phytochemical Analysis 

#### Identification and Quantification of Polyphenolic Compounds by HPLC-DAD-ESI MS

The phenolic compounds of the *T. officinale* extracts were determined as previously described [23,24] with some modifications. Prior to LC analysis, the lyophilized extract was dissolved in MeOH. Chlorogenic acid was used for phenolic acid quantification, and results were expressed as mg chlorogenic acid equiv./g of dry plant material (mg CA/g d.w.) [25].

### 2.4. Animals and Experimental Design

The experiments were carried out on adult male Albino Wistar rats (strain Crl: WI), weighing 200–250 g, bred in the Animal Facility of Iuliu Hațieganu University of Medicine and Pharmacy, Cluj-Napoca as previously described [23]. Animals were randomly divided into 6 groups (*n* = 5): Control group with no disease and no treatment, acute on chronic liver failure (ACLF) group, ACLF with Silymarin pretreatment (ACLF-SYL) group (200 mg/kg b.w./day) [26], ACLF groups with TOERE pretreatment in three doses, respectively ACLF-TO200 (200 mg dry plant material/kg b.w./day), ACLF–TO100 (100 mg dry plant material/kg b.w./day), and ACLF-TO50 (50mg dry plant material/kg b.w./day). The daily dose of TOERE has been dissolved in corn oil (1ml/day/animal). All the procedures performed on laboratory animals, comply with the Directive 2010/63/EU, and Romanian national law 43/2014 for animal protection used for scientific purposes. The project was approved by the Veterinary Sanitary Direction and Food Safety Cluj-Napoca as previously described (no. 19/ 13.12.2016) [23].

The ACLF rat model was induced by human serum albumin (HSA), d-galactosamine (D-Gal), and lipopolysaccharide (LPS) as previously described [27,28]. Silymarin (SYL) or TO have been administrated per os (p.o.) by gavage for 7 days. The ACLF group was pretreated for 7 days with physiological saline (1 mL/day/animal). After completing the treatments, on day 8 in the ACLF, ACLF-TO200, ACLF-TO100, ACLF–TO50, and ACLF-SYL groups ACLF was induced by the intraperitoneal injection (i.p.) of d-galactosamine (D-Gal) (400 mg/kg b.w.) and lipopolysaccharide (LPS) (100 μg/kg b.w.) [27,29]. Six hours after ACLF induction the rats were anesthetized with ketamine (60 mg/kg b.w.) and xylazine (15 mg/kg b.w.), blood was withdrawn by retro-orbital puncture, serum was separated by centrifugation, and stored at −80 °C until use. At the end of the experiment, under general anesthesia animals were killed by cervical dislocation and liver biopsy was harvested from each animal. The experiments were performed in triplicate.

### 2.5. Biochemical Serum Analysis 

The hepatic injury was evaluated with conventional serum liver markers: serum aspartate aminotransferase (AST), alanine aminotransferase (ALT), total bilirubin (BT), alkaline phosphatase (ALP), and gamma glutamate transferase (GGT) as previously described [23]. Oxidative stress associated with liver injury was evaluated by measuring serum total oxidative status (TOS), total antioxidant reactivity (TAR), oxidative stress index (OSI), malondialdehyde (MDA), total thiols (SH), total nitrites, and nitrates (NOx) and 3-nitrotyrosine (3NT) levels as previously described [23,30,31]. Renal failure induced by ACLF was diagnosed with creatinine and urea. 

### 2.6. Histological Assessment 

For the histological analysis two liver fragments were collected from the left lateral and right medial lobes [32], fixed in 10% phosphate-buffered formalin for 24 h, and routinely processed and embedded in paraffin wax. From each tissue fragment, two serial sections of 3 µm were stained with hematoxylin and eosin (H&E). The hepatic parenchyma was histologically assessed for intralobular and periportal degeneration/necrosis, portal inflammation, and fibrosis, and the Histological Activity Index (HAI) was calculated [33,34].

### 2.7. Immunohistochemical Analysis of 3-Nitrotyrosine 

For the immunohistochemical analysis of 3NT, the paraffin sections were dewaxed in xylene, followed by rehydration in decreasing the concentration of alcohol. Sodium citrate buffer (pH = 6) was used for epitope retrieval and endogenous peroxidase was blocked with peroxidase for 5 min. The primary mouse monoclonal [clone 39B6] antibody to 3NT was diluted in 1% PBS-BSA (bovine serum albumin) at 1:200, and maintained overnight at 4 °C in a humid chamber, followed by placing the secondary antibody. The reaction was visualized using 3,3’-diaminobenzidine. Finally, the sections were counterstained with Mayer’s hematoxylin. The positive reaction was given by the brown labeling of the hepatocytes. Immunopositivity for 3NT was evaluated and scored, as follows: grade 0, no staining; grade 1, positive staining in less than 10% of hepatocytes/10 high power fields; grade 2, positive staining in more than 10% but less than 50% of hepatocytes/10 high power fields; grade 3, positive staining of more than 50% of hepatocytes/10 high power fields [35].

The sections were independently examined by two pathologists (MT and CT) using a light Olympus BX-41 microscope, and a multi-head microscope Zeiss Axio Scope A1 (Carl Zeiss Microscopy GmbH, Germany). When there was a divergence of opinion, an agreed diagnosis was reached by a simultaneous evaluation in a multi-head microscope Zeiss Axio Scope A1 (Carl Zeiss Microscopy GmbH, Germany). The photomicrographs were taken using an Olympus SP 350 digital camera and Stream Basic imaging software (Olympus Corporation, Tokyo, Japan).

### 2.8. Statistical Analysis

All results were expressed as mean ± standard deviation (SD) whenever data were normally distributed. Comparisons between the different experimental groups were performed using the one-way ANOVA test and the post hoc Bonferroni–Holm test. The correlations analysis was performed with the Pearson test. Values of *p* < 0.05 were considered statistically significant. The analysis was performed using IBM SPSS Statistics, version 20 (SPSS Inc. Chicago, IL, USA).

## 3. Results

### 3.1. Phytochemical Analysis 

**Figure 1:**
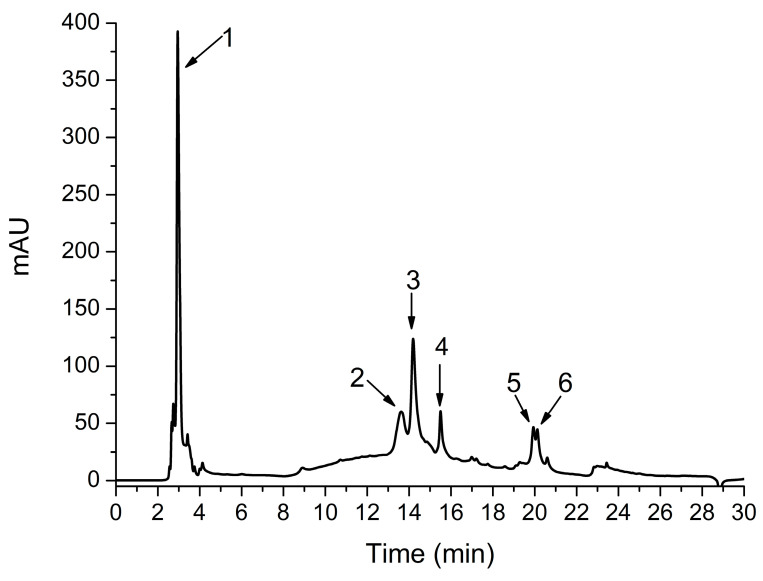
Chromatogram obtained by HPLC-DAD-ESI MS analysis of a *Taraxacum officinale* root extract at 340 nm. For peak assignments, see Table 1.

In our study, HPLC-DAD-ESI MS identified significant concentrations of hydroxybenzoic, caffeic, and chicoric acids (Figure 1, Table 1). 

### 3.2. Biochemical Serum Analysis 

The hepatic injury was evaluated by measuring liver markers (AST, ALT, ALP, GGT, TB) [36] (Table 2). ACLF induction by D-Gal-LPS caused a severe increase of the liver markers than in Control animals (*p* < 0.001). Administration for a week of three different doses of TOERE or SYL in ACLF animals significantly prevented severe ACLF-induced increase of the AST, ALT, ALP, GGT, and TB (*p* < 0.001). Furthermore, the TOERE effect was dose-dependent, with the 100 mg TOERE/kg b.w./day concentration having the best inhibitory effect. In ACLF-TO200 and ACLF-TO100 groups TOERE hepatoprotective effects were better than in ACLF-SYL animals (*p* < 0.01) (Table 2).

Considering that in ACLF kidneys are the most affected organs, in a study of a plant with possible hepatoprotective use in ACLF it is also important to determine the nephroprotective activity. ACLF induction by D-Gal- LPS caused a severe increase of creatinine and urea (*p* < 0.001). Renal dysfunction tests were positively correlated with the liver markers (r = 0.6–0.9) in ACLF animals. The treatments of ACLF rats with TOERE or SYL caused a smaller increase of serum creatinine and urea after ACLF induction (*p* < 0.001). SYL effect was comparable to that from ACLF-TO200 and ACLF-TO100 groups, but better than that from ACLF-TO50 animals (Table 2). 

In our study systemic oxidative stress was also evaluated. Compared to the control, serum TOS, OSI, and MDA were elevated in ACLF animals (*p* < 0.001. The treatment with TOERE or SYL reduced TOS, OSI and MDA increase after ACLF induction (*p* < 0.01). TOERE effect on the oxidative stress was dose-dependent, the higher concentration having the best antioxidant effect (Table 3).

In ACLF rats NOx and 3NT were also increased (*p* < 0.001). TOERE pretreatments prevented NOx and 3NT elevation (*p* < 0.001) after ACLF induction in a dose-dependent way, with a higher concentration having a better inhibitory activity. SYL was also a good inhibitor of NO production and peroxidation in ACLF animals (*p* < 0.001), but the effect was smaller than that of TOERE (Table 3). 

Additionally, serum antioxidative activity was evaluated by measuring TAR and SH. TAR was not influenced by ACLF induction (*p* > 0.05). A depletion in the level of SH was observed in ACLF rats (*p* < 0.01), and the treatment with TO or SYL prevent SH reduction (*p* < 0.05) after ACLF induction. SYL has a better effect than TO on SH (Table 3).

### 3.3. Histological Assessment

**Figure 2:**
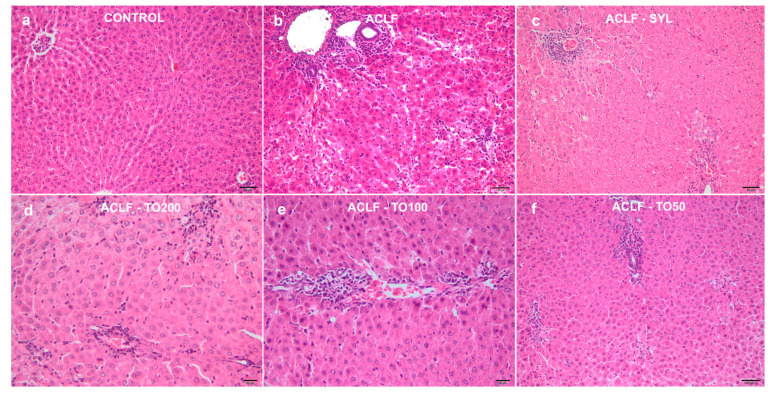
Photomicrographs of the liver tissues from the control and experimental animals. H&E stain: (**a**). Control; (**b**). ACLF; (**c**). ACLF-SYL; (**d**). ACLF-TO200; (**e**). ACLF-TO100; (**f**). ACLF-TO50; Bar = 50 µm (**a**–**c**,**f**) and 20 µm (**d**,**e**). ACLF—acute on chronic liver failure; ACLF-SYL—acute on chronic liver failure pretreated with 200 mg silymarin/kg b.w./d; ACLF-TO200—acute on chronic liver failure pretreated with 200 mg TOERE/kg b.w./day; ACLF-TO100—acute on chronic liver failure pretreated with 100 mg TOERE/kg b.w./day; ACLF-TO50—acute on chronic liver failure pretreated with 50 mg TOERE/kg b.w./day.

In the livers of the Control group, no significant structural changes were observed (Figure 2a).

The highest histological scores were identified in the livers of the ACLF group (*p* < 0.001) (Table 4). The changes were represented by congestion, hemorrhages, multifocal to coalescing areas of coagulative necrosis, randomly distributed within the hepatic lobules or centered on periportal regions and associated with severe and mixed inflammatory infiltrates. The portal spaces were also affected and expanded by fibrosis, bile duct hyperplasia, and large numbers of inflammatory cells, predominated by small lymphocytes and macrophages (Figure 2b). The microscopical examination of the livers from the group ACLF-SYL group (Figure 2c) revealed the lowest histological scores if compared to ACLF, ACLF-TO200, ACLF-TO100, and ACLF-TO50 (*p* < 0,001). Compared to the untreated ACLF group, in ACLF-TO200 (Figure 2d), ACLF-TO100 (Figure 2e), and ACLF-TO50 (Figure 2f) animals, the hepatic injuries were significantly reduced by the TOERE pretreatments (*p* < 0.001), with no important differences between different TOERE doses (*p* > 0.05). Liver necroinflammatory scores and serum liver tests were positively correlated.

### 3.4. 3-Nitrityrosine Evaluation

**Figure 3:**
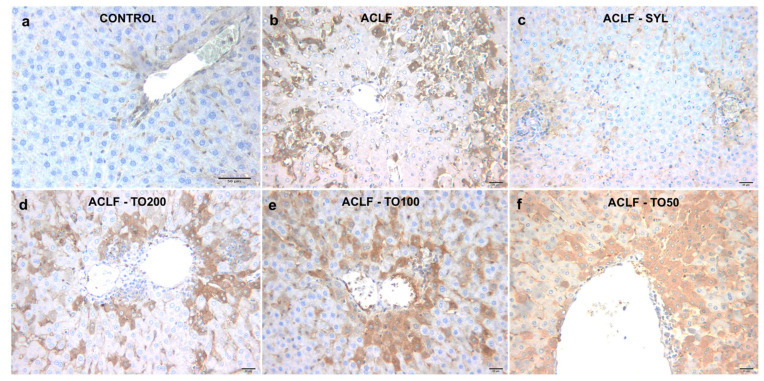
Immunohistochemical expression of 3-nitrotyrosine (3-NT) in liver tissues from the control and experimental animals: (**a**). Control; (**b**). ACLF; (**c**). ACLF-SYL; (**d**). ACLF-TO200; (**e**). ACLF-TO100; (**f**). ACLF-TO50; Bar = 50 μm (**a**) and 20 μm (**b**–**f**). ACLF-TO200—acute on chronic liver failure pretreated with 200 mg TOERE/kg b.w./day; ACLF-TO100—acute on chronic liver failure pretreated with 100 mg TOERE/kg b.w./day; ACLF-TO50—acute on chronic liver failure pretreated with 50 mg TOERE/kg b.w./day; ACLF-SYL—acute on chronic liver failure pretreated with 200 mg silymarin/kg b.w./d.

3-NT immunoexpression was negative in the livers of the control group (Figure 3a). A marked hepatocellular immunoexpression of 3-NT with a diffuse or mediolobular pattern was found in all liver samples from the ACLF group (Figure 3b) (*p* < 0.01). In the group ACLF-SYL, the 3-NT expression was reduced compared to the ACLF group (*p* < 0.01), being mainly limited to hepatocytes near the portal spaces (Figure 3c) (Table 4).

As compared to the ACLF group, the expression of 3NT was lower in liver biopsies from TOERE treated animals, particularly in the ACLF-TO200 (Figure 3d) and ACLF-TO100 (Figure 3e) (*p* < 0.01) groups. The expression of 3NT in the ACLF-TO50 group (Figure 3f) was higher compared to the other treated groups. SYL effect on 3NT expression was better than that of TOERE (*p* < 0.05) (Table 4).

The correlation between the histological scores and biochemical tests were also analyzed. In ACLF, ACLF-TO200, ACLF-TO100, ACLF-TO50, and ACLF-SYL groups all histopathological scores were positively correlated with liver, renal, and oxidative stress markers.

## 4. Discussion

In the current study, D-Gal-LPS-induced ACLF in rats with HAS-induced chronic liver failure triggered an immune-mediated liver injury with pathological serum liver marker tests and histological liver changes. The liver injuries were also associated with renal failure and systemic oxidative stress. A seven days pretreatment with TOERE reduced ACLF induced liver injury. The protecting effect of TOERE can be attributed, at least in part, to the reduction of the oxidative stress associated with immune liver injury in D-Gal-LPS-induced ACLF. Depending on the dose, the hepatoprotective effect of TOERE was similar or lower than that of SYL, an already used hepatoprotective drug.

By analyzing the TOERE extract, other studies identified sesquiterpenes, various triterpenes, phenolic compounds, and phytosterols. Our previous phytochemical analysis [23] showed that the tested TO root extract had a lower TPC than in Aremu et al.’s analysis of TO root extract (1.14 ± 0.01 mg/100 GAE/mg extract) and TO leaf extract (4.35 ± 0.15 mg GAE/mg extract) [37], but higher than in the TO aerial part extract (15.50 mg GAE/g d.w.) [38].

The HPLC-DAD-ESI MS analysis of our TO root extract identified, caffeic acid, chicoric acid, as previously described [23], plus feruloylquinic acid, dicaffeoylquinic acid, and dicaffeoylquinic acid isomer. All these compounds have anti-inflammatory and antioxidant properties [11,23,39,40,41]. The antioxidant activity of the TOERE measured by DPPH and FRAP tests was proved in our previous study [23].

Because the chemical composition correlates with the pharmacological effects, TO extracts from different plant parts had different activities. Several studies demonstrated that the TO roots extract reduces alcohol-induced oxidative stress, TO leaf extract alleviates high-fat diet-induced nonalcoholic fatty liver, and TO flower extract can scavenge reactive oxygen species [22]. Similar to other studies [16,42,43], and based on the evidence of the phytochemical analysis results, our TO root extract can be considered a good natural antioxidant candidate. These results encouraged us to continue by testing the in vivo hepatoprotective and antioxidant effects of TOERE in an experimental ACLF.

For ACLF experimental model, first HAS administration in rats caused an immune liver injury and fibrosis, and then LPS stimulated liver macrophages leading to hepatic necro-inflammatory change. D-Gal, an amino sugar metabolized selectively by the hepatocytes, in a few hours potentiated the hepatotoxic effect of LPS by inhibiting mRNA and protein synthesis, leading to acute hepatitis [28]. The hepatoprotective effect of the TOERE was evaluated by using serum liver markers and liver histological analysis [2,3]. AST and ALT elevation reflects generalized damage to hepatocytes, TB increase reflects liver metabolism, ALP and GGT elevation reflects cholestasis [20,43,44]. In ACLF rats, liver markers were consistent with hepatocytes injury, cholestasis and, lower liver metabolism, demonstrating that an ACLF model was successfully induced [27]. The preliminary tests evaluating the TOERE effect on negative control animals indicated that the product had no significant activity on the healthy liver and oxidative stress (see supplemental data). In ACLF TO pretreatment reduced liver markers, suggesting that TOERE may prevent severe ACLF and by that to reduce the mortality due to ACLF. ACLF-TO100 group had the best hepatoprotective effect. In a previous study, we also evaluated in the TO root extract some phytosterols with anti-inflammatory and antioxidative properties [23]. It was demonstrated that due to their structural similarity with cholesterol, phytosterols are prone to be oxidized and transformed into oxyphytosterols [45,46,47], and from antioxidants to become pro-oxidants. By lowering the dose of TO from 200 to 100mg dry plant material/kg b.w./day, phytosterol reduction may be involved in the better hepatoprotective effect of ACLF-TO100 than of ACLF-TO200.

Liver injury diagnosed by serum liver tests was further confirmed by histopathological characteristics. In a normal liver, there is a hypoimmune response, and in chronic liver inflammation there is a high cellular recruitment, extended tissue damage, and the repair process leads to tissue remodeling, fibrosis, and liver dysfunction [41,48,49]. Fibrosis represents a key characteristic of progression towards liver cirrhosis and hepatic failure. In the present work liver biopsy of the ACLF animals showed extended necro-inflammatory changes, fibrosis, and bile ducts hyperplasia. In ACLF rats, like previously observed, histopathological scores increased due to the ongoing inflammation activation and the direct cytotoxic effect of cell death products [50]. Pretreatment with our TOERE in ACLF had hepatoprotective activity by reducing liver necro-inflammatory changes, with no important effect on liver fibrosis. 

Under physiological conditions, free radicals are scavenged by antioxidant mechanisms. If there is an excess of free radicals or if there is a deficiency of antioxidants, oxidative stress will build up and will cause oxidative damage of lipids, proteins, and DNA [2,43,49]. In D-Gal-LPS-induced ACLF, immune-induced liver injury triggered an important liver inflammatory response and systemic oxidative stress, with high serum TOS and OSI, along with increased production of MDA, NOx, and 3NT. 

Many studies correlated the hepatoprotective activity of the medicinal plant extracts with the antioxidant compounds from these plants [51]. Moreover, other experimental studies demonstrated that the polyphenolic compounds isolated from TO extracts had a hepatoprotective effect by reducing oxidative stress through direct free radical scavenging activity, metal ions chelation, and regeneration of the membrane-bound antioxidants [40,49]. In the present work, TOERE decreased serum TOS, OSI, and MDA levels in a dose-dependent way. MDA reduction was relevant because recently lipid peroxidation was considered a vital process in chronic liver diseases [44]. TOERE did not affect TAR, and SH was just slightly increased, indicating that this extract reduced systemic oxidative stress mainly by scavenging the oxidants and less by increasing the antioxidant capacity. 

In mammals there are three NO synthase (NOS) isoenzymes that are involved in NO synthesis: neuronal (nNOS/NOS-1), inducible (iNOS/NOS-2), and endothelial (eNOS/NOS-3). Inflammatory stimuli up-regulate iNOS, and excessively generate NO induces nitrosative and oxidative damage. In liver injury, NO can be produced by hepatocytes, Kupffer cells, hepatic stellate cells (HSCs), and hepatic sinusoidal endothelial cells. It was observed that NO may have a dichotomous effect on liver disease, respectively in chronic liver diseases NO can promote HSC apoptosis, but in acute liver diseases, NO may increase liver damage. In LPS-treated rats, the marked hepatocellular immunoexpression of 3NT indicated that the iNOS-ROS cycle augments liver injury [52]. In this study, we found that liver 3NT was down-regulated following treatment with TOERE, suggesting that TOERE may reduce liver inflammatory responses and oxidative stress by reducing NO production through the inhibition of iNOS gene expression. These properties of TOERE may be explained by the high content of antioxidant phytochemicals.

Only iNOS and eNOS were highly expressed in acute liver failure (ALF) liver tissue, causing plasma NO elevation, and in humans increased plasma NO levels were correlated to the clinical severity of ALF [52]. In D-Gal-LPS-induced ACLF elevation of serum NOx and 3NT confirmed excessive NO synthesis due to the severe liver injury and inflammation. The treatment with TOERE reduced the serum NOx and 3NT, indirectly indicating that TOERE had a significant inhibitory effect on systemic NO production.

Because systemic oxidative stress markers reduction was correlated with serum liver markers and liver histopathological scores improvement, we concluded that TOERE lowered liver injury by reducing oxidative stress. Like in other studies [10], it was found that the antioxidant effect of TOERE was dose-dependent, the higher extract concentration had better antioxidant activity due to the high concentration of antioxidant ingredients. 

According to the World Gastroenterology Organization ACLF is characterized by acute liver failure and one or more extrahepatic organ failure because in ACLF liver inflammation may trigger systemic inflammation. The kidneys are the most commonly affected organs and renal failure range from acute kidney injury (AKI) to acute-on-chronic kidney failure [2,3]. Therefore, acute kidney injury (AKI) was tested as a major criterion in ACLF severity grading [53]. In ACLF two subgroups of secondary renal dysfunctions with different pathophysiology and prognosis can be associated. One is the hepatorenal syndrome-acute kidney injury (HRS-AKI), a reduction of kidney function without parenchymal damage caused by prerenal insults such as hypovolemia. The other one is the non–HRS-AKI, induced by a renal insult such as inflammatory tubular injury in sepsis, bile acid nephropathy, and drug-induced tubular damage [54]. In ACLF liver protein synthesis lowers and may cause complications like coagulopathy, hemodynamic instability, jaundice, hepatic encephalopathy, hepatorenal syndrome, and sepsis [50]. At the same time, the systemic inflammatory response may also cause an inflammatory kidney injury with anon–HRS-AKI. Moreover, in experimental ACLF proinflammatory cytokines and LPS can cause directly renal tubular injury with cell apoptosis. Intrahepatic cholestasis from ACLF with increased serum bilirubin and bile acids may induce renal injury due to the direct renal toxicity and by tubular obstruction [54]. In our study, in ACLF animals creatinine and urea reached AKI levels, and there was a positive correlation between serum creatinine and urea, liver biopsy scores, and serum liver test. TOERE and SYL pretreatments reduced serum creatinine and urea in a dose-dependent way, indicating that in ACLF animals TOERE hepatoprotective activity is associated with a nephroprotective effect.

Lately, SYL has been used as a hepatoprotective agent due to its antioxidant and anti-inflammatory effects [55]. A finding of the study was that in experimental ACLF TO roots extract effects on serum liver markers were better than those of SYL, and SYL caused a higher reduction of the liver histological scores and 3NT immunoexpression. These differences suggested that TOERE better prevented acute liver injury and SYL reduces more the chronic response to liver injury. Even when SYL can reduce oxidative stress by scavenging ROS, by inhibiting ROS production [56], and by activating antioxidant enzymes [55], in our study it had a lower systemic antioxidant activity than TOERE.

## 5. Conclusions

This report highlights the hepatoprotective and nephroprotective effects of an ethanolic TO root extract on D-Gal-LPS-induced ACLF. The mechanism proposed is the antioxidant activity of the bioactive components of the TOERE. These findings suggest for the first time that TOERE may be a potential preventive therapeutic agent for the severe liver and renal inflammatory injury associated with ACLF. These observations are important considering that ACLF has a high mortality rate. Further studies and clinical trials are required to fully elucidate the beneficial effects of TO root extract supplementation to prevent ACLF.

## Figures and Tables

**Table 1:** Identification and quantification of *Taraxacum officinale* root extract polyphenols from hydroxybenzoic and hydroxycinnamic acids groups.

No	Retention Time R_t_ (min)	UV λ_max_ (nm)	[M+H]^+^ (m/z)	Tentative Identification	Concentration * mg CA/ g TOERE
1	2.95	270	138	Hydroxybenzoic acid	3.65 ± 0.15
2	13.62	320	181, *163*	Caffeic acid	1.09 ± 0.02
3	14.19	322	475, *312*	Chicoric acid	1.95 ± 0.15
4	15.50	322	369	Feruloylquinic acid	0.6 ± 0.08
5	19.93	322	516, *181,163*	Dicaffeoylquinic acid	0.53 ± 0.04
6	20.12	322	516, *181,163*	Dicaffeoylquinic acid isomer	0.4 ± 0.03

* mg CA/g TOERE-chlorogenic acid equiv. mg/g *Taraxacum officinale* ethanolic root extract. Values are the mean ± SD (*n* = 3).

**Table 2:** Liver and renal screening tests of the study groups.

Groups	AST (U/L)	ALT (U/L)	TB (mg/dL)	ALP (mg/dL)	GGT (mg/dL)	Urea (mg/dL)	CR (mg/dL)
ACLF-TO200	81.12 ^a^ ± 5.27	71.64 ^a,b,c^ ± 11.32	2.27 ^a,b,c^ ± 0.37	328.45 ^a,b^ ± 14.72	60.42 ^a,b,c^ ± 9.20	67.14 ^a,b,c^ ± 4.21	1.75 ^a,b^ ± 0.21
ACLF-TO100	82.14 ^a,b,c^ ± 4.20	54.08 ^b,c^ ± 12.37	1.30 ^b,c^ ± 0.27	310.38 ^a,b,c^ ± 11.19	49.97 ^b,c^ ± 8.37	78.93 ^a,b^ ± 5.18	1.78 ^a,b^ ± 0.14
ACLF-TO50	84.24 ^a,b,c^ ± 8.06	144.93 ^a,b,c^ ± 19.79	2.02 ^a,b^ ± 0.51	329.61 ^a,b^ ± 37.89	107.34 ^a,b,c^ ± 18.33	110.30 ^a,b,c^ ± 7.89	2.15 ^a,b^ ± 0.40
ACLF-SYL	126.37 ^a,b^ ± 6.58	111.67 ^a,b^ ± 13.04	2.44 ^a,b^ ± 0.13	332.59 ^a^ ± 29.20	74.51 ^a,b^ ± 9.86	81.25 ^a,b^ ± 12.15	2.02 ^a,b^ ± 0.29
ACLF	222.65 ^a,c^ ± 11.08	174.08 ^a,c^ ± 15.16	3.74 ^a,c^ ± 0.53	358.94 ^a,c^ ± 13.55	117.71 ^a,c^ ± 15.47	255.49 ^a,c^ ± 19.48	3.53 ^a,c^ ± 0.28
Control	35.04 ± 6.63	47.55 ± 10.08	1.01 ± 0.11	263.75 ± 15.20	44.31 ± 4.58	39.16 ± 2.71	0.57 ± 0.04

Results are expressed as mean ± SD. Values are expressed as mean ± SD (*n* = 5). ^a^ *p* ˂ 0.05, versus Control; ^b^ *p* ˂ 0.05, versus ACLF; ^c^ *p* ˂ 0.05, versus SYL. AST—aspartate aminotransferase; ALT—alanine aminotransferase; TB—total bilirubin; ALP—alkaline phosphatase; GGT—gamma-glutamyltransferase; CR—creatinine; ACLF-TO200- acute on chronic liver failure pretreated with 200 mg TOERE/kg b.w./day; ACLF-TO100—acute on chronic liver failure pretreated with 100mg TOERE/kg b.w./day; ACLF-TO50—acute on chronic liver failure pretreated with 50 mg TOERE/kg b.w./day; ACLF-SYL—acute on chronic liver failure pretreated with 200 mg silymarin/kg b.w./d; ACLF—acute on chronic liver failure; Control—negative control.

**Table 3:** Oxidative stress tests of the study groups.

Groups	TOS (µM H_2_O_2_/L)	TAR (mM TROLOX/L)	OSI	MDA (nM/L)	NOx (µM/L)	3NT (nmol/L)	SH (mM GSH/L)
ACLF-TO200	30.61 ^a,b,c^ ± 6.85	1.088 ± 0.001	31.57 ^a,b,c^ ± 6.13	3.05 ^a,b,c^ ± 0.28	21.92 ^b,c^ ± 3.74	769.36 ^a,b,c^ ± 78.46	0.48 ^a,b^ ± 0.03
ACLF-TO100	35.50 ^a,b,c^ ± 7.27	1.089 ± 0.001	31.17 ^a,b,c^ ± 4.84	3.67 ^b^ ± 0.59	25.76 ^a,b,c^ ± 4.50	768.66 ^a,b,c^ ± 69.75	0.48 ^a,b^ ± 0.08
ACLF-TO50	40.45 ^a,b,c^ ± 8.46	1.089 ± 0.001	31.82 ^a,b,c^ ± 9.39	3.95 ^a,b^ ± 0.47	30.52 ^a,b,c^ ± 7.60	820.20 ^a,b,c^ ± 48.43	0.48 ^a,b^ ± 0.02
ACLF-SYL	36.41 ^a,b^ ± 7.75	1.092 ± 0.003	37.03 ^a,b^ ± 8.27	3.83 ^a,b^ ± 0.34	36.56 ^a,b^ ± 6.76	971.07 ^a,b^ ± 68.34	0.52 ^a,b^ ± 0.02
ACLF	47.98 ^a,c^ ± 7.95	1.089 ± 0.001	40.40 ^a,c^ ± 8.60	5.37 ^a,c^ ± 0.08	51.49 ^a,c^ ± 7.32	1053.99 ^a,c^ ± 91.15	0.40 ^a,c^ ± 0.03
Control	21.18 ± 1.72	1.089 ± 0.001	21.59 ± 4.61	3.57 ± 0.36	19.98 ± 1.99	480.45 ± 56.62	0.59 ± 0.01

Results are expressed as mean ± SD. ^a^ *p* ˂ 0.05, versus Control; ^b^ *p* ˂ 0.05, versus ACLF; ^c^ *p* ˂ 0.05, versus SYL. TOS—total oxidative status; TAR—total antioxidant reactivity; OSI—oxidative stress index; NOx—nitric oxide; 3NT—3-nitrotyrosine; MDA—malondialdehyde; SH—total thiols; ACLF-TO200—acute on chronic liver failure pretreated with 200 mg TOERE/kg b.w./day; ACLF-TO100—acute on chronic liver failure pretreated with 100 mg TOERE/kg b.w./day; ACLF-TO50—acute on chronic liver failure pretreated with 50 mg TOERE/kg b.w./day; ACLF-SYL—acute on chronic liver failure pretreated with 200 mg silymarin/kg b.w./d; ACLFacute on chronic liver failure; Control—negative control.

**Table 4:** Histological and IHC scores of the liver biopsies.

Groups	Portal Inflammation	Periportal Degeneration/ Necrosis	Intralobular Degeneration/ Necrosis	Fibrosis	HAI	3NT
ACLF-TO200	1.60 ^a,b,c^ ± 0.89	2.20 ^a,b,c^ ± 0.10	1.00 ^a,b,c^ ± 0.01	1.20 ^a,c^ ± 0.10	5.80 ^a,c^ ± 1.92	1.40 ^a,b,c^ ± 0.55
ACLF-TO100	2.20 ^a,b,c^ ± 0.10	2.60 ^a,b,c^ ± 0.89	1.40 ^a,b,c^ ± 0.89	1.00 ^a,c^ ± 0.10	7.20 ^a,b,c^ ± 1.10	1.40 ^a,b,c^ ± 0.55
ACLF-TO50	2.60 ^a,b,c^ ± 0.89	2.20 ^a,b,c^ ± 1.10	2.20 ^a,b,c^ ± 1.10	1.00 ^a,c^ ± 0.10	8.00 ^a,b,c^ ± 1.41	1.80 ^a,b,c^ ± 0.45
ACLF-SYL	1.00 ^a,b^ ± 0.00	0.60 ^a,b^ ± 0.55	0.80 ^a,b^ ± 0.45	0.40 ^a,b^ ± 0.55	2.80 ^a,b^ ± 0.45	1.20 ^a,b^ ± 0.45
ACLF	3.60 ^a,c^ ± 0.55	4.80 ^a,c^ ± 0.84	3.60 ^a,b^ ± 0.55	1.00 ^a,b^ ± 0.10	12.80 ^a,b^ ± 1.64	2.40 ^a,b^ ± 0.55
Control	0.40 ± 0.55	0.00 ± 0.00	0.00 ± 0.00	0.00 ± 0.00	0.20 ± 0.45	0.00 ± 0.00

Results are expressed as mean ± SD. ^a^ *p* ˂ 0.05, versus Control; ^b^ *p* ˂ 0.05, versus ACLF; ^c^ *p* ˂ 0.05, versus SYL; ACLF-TO200—acute on chronic liver failure pretreated with 200 mg TOERE/kg b.w./day; ACLF-TO100—acute on chronic liver failure pretreated with 100mg TOERE/kg b.w./day; ACLF-TO50—acute on chronic liver failure pretreated with 50 mg TOERE/kg b.w./day; ACLF-SYL—acute on chronic liver failure pretreated with 200 mg silymarin/kg b.w./d; ACLF—acute on chronic liver failure; Control—negative control; HAI—histological activity index; 3NT—3-nitrotyrosine.

## Data Availability

Not applicable.
